# Early Intervention after Rape to prevent post-traumatic stress symptoms (the EIR-study): an internal pilot study of a randomized controlled trial

**DOI:** 10.1186/s40814-024-01541-0

**Published:** 2024-09-02

**Authors:** Tina Haugen, Joar Øveraas Halvorsen, Oddgeir Friborg, Paul Jarle Mork, Gustav Mikkelsen, Berit Schei, Cecilie Hagemann

**Affiliations:** 1 Department of Clinical and Molecular Medicine, Norwegian University of Science and Technology (NTNU), Trondheim, Norway; 2 Department of Mental Healthcare, St. Olavs Hospital, Trondheim University Hospital, Trondheim, Norway; 3 Department of Psychology, Norwegian University of Science and Technology (NTNU), Trondheim, Norway; 4 Department of Psychology, The Arctic University of Norway (UiT), Trondheim, Norway; 5 Department of Public Health and Nursing, Norwegian University of Science and Technology (NTNU), Trondheim, Norway; 6 Department of Clinical Chemistry, St. Olavs Hospital, Trondheim University Hospital, Trondheim, Norway; 7 Department of Obstetrics and Gynecology, St. Olavs Hospital, Trondheim University Hospital, Trondheim, Norway

## Abstract

**Background:**

Rape is one of the trauma incidents with the highest risk of subsequent post-traumatic stress disorder. Early interventions, such as prolonged exposure therapy (PE), have shown promise in preventing PTSD following a sexual assault. The primary objective of this internal pilot trial was to examine the feasibility of the EIR study protocol, which used modified prolonged exposure therapy (mPE) as a preventive intervention after rape.

**Methods:**

This parallel two-arm clinical pilot study involved three sexual assault centers (SACs) in Trondheim, Oslo, and Vestfold, with data collected between June 2022 and March 2023. Women seeking assistance at one of these three SACs within 72 h after rape or attempted rape received acute medical treatment and forensic examinations. Women who wanted further psychosocial treatment were, if eligible and consenting, recruited to complete baseline assessments and a clinical interview before being randomized to one of two study arms. The intervention group prescribed up to five sessions of modified PE (mPE) in addition to treatment as usual (TAU), starting within the first 14 days after the rape incident, followed by weekly sessions. The other group received TAU.

The present pilot evaluation is based on 22 participants, i.e., nine mPE + TAU and 13 TAU alone. Primary outcomes were predefined progression criteria regarding recruitment, retention, intervention implementation, a harm reporting system, and applying biological measurements and actigraphy.

**Results:**

During the 6-month recruitment period, 235 women visited the three SACs. After eligibility screening and consent, 22 (9.4%) women were randomized. Three months later, 14 (63.6%) participants completed the final assessments. Intervention implementation was successful using trained SAC personnel to deliver mPE. The harm reporting system was used according to the study’s plan, and adverse and serious adverse events were detected during the trial. The biological measurements and actigraphy had substantial missing data but were still considered usable for statistical analyses.

**Conclusion:**

It may be feasible to conduct a full-scale RCT of early intervention after rape by comparing mPE + TAU to TAU alone. Minor design refinements were made to the protocol to enhance the main study outcome.

**Trial registration:**

ClinicalTrials.gov Identifier: NCT05489133. Registered on 15 July 2022, retrospectively.

## Key messages regarding feasibility

- What uncertainties exist regarding the feasibility? Uncertainties regarding the feasibility of conducting a randomized controlled trial in a population subjected to recent rape in a Norwegian setting were investigated, as well as the recruitment of sexual assault centers (SACs) as study sites. There were also uncertainties regarding the use of trained SAC personnel instead of experienced specialists to implement a new intervention, and the degree of adherence to the protocol. The application of a large measurement battery (i.e., questionnaires on sensitive topics, biological samples, and actigraphy) needed also to be tested.
- What are the key feasibility findings? The key feasibility findings were that the recruitment rate was slow in the beginning, the intervention was safe and acceptable for participants, the training program for SAC personnel was successful, and participants were motivated to collect biological data and actigraphy.
- What are the implications of the feasibility findings for the design of the main study? The findings demonstrated that it is feasible to proceed with the main RCT, with minor adjustments, especially regarding recruitment.

## Background

Post-traumatic stress disorder (PTSD) is the most frequent psychopathological consequence following traumatic incidents. Compared to other traumas, interpersonal sexual trauma, and especially rape, is associated with the highest rates of PTSD [1, 2]. PTSD further heightens the risk of comorbid health issues such as mood or anxiety disorders [1], alcohol or substance abuse disorder, and suicide [3, 4]. Survivors of rape also report more somatic illnesses, such as chronic abdominal, pelvic, and vulvar pain, and sexual dysfunction [5–8]. The hormone cortisol is involved in the regulation of stress, and several studies have used cortisol levels as a predictor of the development of PTSD. The role of cortisol after trauma is however ambiguous; stress is normally associated with increased secretion of cortisol, but in cases of prolonged stress exposure and traumatization, decreased cortisol secretion is shown [9].

A recent population study in Norway reported that 22% of women and 3% of men had experienced a lifetime incident of forceable or incapacitated rape [10]. This underscores the need for effective interventions to prevent the negative health effects of rape, including severe and chronic PTSD. Research is focused on preventive programs and effective interventions to mitigate these outcomes.

According to Caplan [11], primary prevention focuses on preventing the onset of mental health issues before they occur by reducing the incidence of new cases. In contrast, secondary prevention focuses on early detection and intervention to address symptoms in their initial stages, to prevent them from escalating into more severe illnesses. In the literature on early intervention, secondary prevention is typically operationalized as interventions administered after the traumatic event but before the development of persistent PTSD, generally within the first months [12].

Currently, there are no widely accepted or reliable predictors for determining who will develop PTSD or respond to early interventions following trauma. The literature reveals significant variability in terms of sample characteristics (e.g., type of trauma and heterogeneity), outcome measures (PTSD diagnosis, post-traumatic stress symptoms, and symptom severity), intervention timing (ranging from a few hours to 3 months post-trauma), intervention dosage (from a single session to 12 sessions), and intervention type (including psychoeducation, video interventions, cognitive therapy with or without exposure, and EMDR). Additionally, the role of natural recovery remains unclear.

The efficacy of prolonged exposure therapy for treating chronic PTSD, including among rape survivors, is well-documented [13–16] and recommended by most international guidelines [17]. Research has focused on whether modified versions of PE can be effective as early interventions to prevent PTSD. Rothbaum and colleagues [18] investigated a modified version of prolonged exposure (mPE) therapy as an early intervention strategy for individuals (*n* = 137) who had suffered different types of traumas (e.g., vehicle accidents, sexual and physical assaults). Their study demonstrated moderate reductions in post-traumatic stress symptoms for those receiving three sessions of mPE, with particularly notable effects among rape victims (*n* = 47). In contrast, a subsequent study by Maples-Keller and colleagues [19] revealed no significant differences in PTSD symptoms between control and intervention groups receiving one or three mPE sessions. The discrepancy with the Rothbaum study could be attributed to differences in sample characteristics, notably the lower proportion of rape survivors in the latter trial. When Larsen et al. [20] evaluated three sessions of mPE in a single-incident trauma sample that did not include rape survivors, they found no significant differences in PTSD symptoms between the mPE and control groups at 1 and 3 months post-injury. As in the Maples-Keller study, the lack of sexual assault cases in their sample might explain the variance in outcomes compared to the Rothbaum study, suggesting that the responsiveness to mPE may vary significantly based on the type of trauma experienced.

Oosterbaan et al. [21] focused on early interventions following sexual assault in their meta-analysis of seven studies. They found that early interventions were safe and significantly reduced PTSD symptom severity compared to standard care, particularly in the long term (2 to 12 months post-intervention). Similarly, Short et al. [12] reviewed 10 studies on the secondary prevention of PTSD following sexual assault, identifying cognitive-behavioral strategies as both safe and effective. They reported a small-to-moderate effect in reducing PTSD and related symptoms, reinforcing the utility of these strategies for recent sexual assault survivors. This finding adds to the growing evidence supporting early, cognitive-behavioral approaches for PTSD prevention.

Bragesjö et al. [22] introduced a novel digital intervention, condensed internet-delivered prolonged exposure (CIPE), for trauma survivors. Their findings showed significant reductions in post-traumatic stress symptoms in the intervention group compared to a waitlist control, with moderate-to-large effect sizes immediately post-intervention and at the 1-month follow-up. The absence of severe adverse events supports the feasibility and safety of digital platforms for delivering timely interventions, which could be especially beneficial in broadening access to care.

In summary, trauma-focused CBT and modified PE show promise for preventing PTSD, but their efficacy appears to be influenced by the nature of the traumatic event and the characteristics of the population. Studies on heterogeneous samples seem to reduce the observed effect. Future research should standardize methodologies and consider the type of trauma to develop more tailored and effective early interventions for PTSD.

### The EIR study

In a sample exclusively consisting of rape survivors, the EIR study aims to investigate the comparative effectiveness of mPE. The study is set against the backdrop of Norway’s specialized sexual assault centers, which were established and expanded since the first one opened in 1986. Today, there are 24 SACs throughout the country, varying in size and structure, located at hospitals or primary care emergency medical wards. SACs operate 24/7, offering comprehensive services such as medical treatment, forensic examinations, and psychosocial support to individuals of all ages and genders who have experienced sexual assault.

Psychosocial support at SACs is tailored to meet individual needs rather than following a standardized approach. However, there is notable variability in the type and quality of psychosocial care provided across different centers, partly due to vague and unclear guidelines outlined in national directives [23]. This lack of clarity contributes to inconsistencies in the support available to survivors at SACs across Norway [24].

Designed as a multicenter randomized controlled add-on superiority trial, the EIR study aims to examine the effects of mPE + TAU compared to TAU alone, on post-traumatic stress symptoms [25]. The hypothesis is that patients receiving mPE + TAU within 2 weeks after rape will have fewer post-traumatic stress symptoms 3 months after rape and that more patients will remain symptom-free at follow-up 6 and 12 months later compared to TAU alone. We also hypothesize that the intervention is safe and can be offered shortly after rape at the SACs and that using SAC personnel trained in the delivery of mPE is viable.

### Aim of the internal pilot study

The objectives were:

1. To determine how many patients consented to participate, completed baseline measures, and were randomized. Given the recruitment process and rate in the internal pilot, is it reasonable to expect that we will meet our estimated sample size within the timeframe of the main trial? If the recruitment rate is too low, what can be done to increase the rate without compromising the main objectives of the EIR study protocol?
2. To evaluate retention, and to what extent participants completed the questionnaires. How many dropped out, when, and for what reasons?
3. The implementation of mPE: Could the mPE intervention be delivered with adherence to the protocol by personnel at the SACs, after receiving proper training from an expert?
4. Detecting harms: How, and to what extent are adverse events and serious adverse events reported by participants and therapists? Are harms related to participating in the study?
5. How many participants are willing to take hair- and saliva samples, and to use actigraphy for 7 days, both at baseline and post-assessments? The logistics of participants returning samples, sending samples from the SAC to the laboratory, does it work according to plan? Is the laboratory able to analyze the self-collected samples?

## Methods

The reporting of this internal pilot study complies with the Consolidated Standards of Reporting Trials (CONSORT) guidelines extended for randomized pilot and feasibility trials [26, 27].

### Trial design

This two-armed parallel design will randomly assign participants to either mPE + TAU or TAU alone in a 1:1 ratio. The mPE participants will be offered the same medical examination and treatment, forensic documentation, and psychosocial follow-up options as the TAU group, with the mPE intervention provided as an additional treatment. Outcome data were collected at baseline, post-treatment, and 3, 6, and 12 months post-trauma.

### Participants and procedures

In total, 22 eligible and consenting women seeking help at one of the three SACs within 72 h after rape or attempted rape were included in the study. The definition of rape or attempted rape for this purpose is “penetration in any body orifice (by penis, finger, foreign body), but also attempted penetration leading to a sufficient mental reaction (helplessness, without control, intense fear”. The initial screening (on age, gender, type of assault, current psychiatric and medical status, etc.) was conducted by senior staff members at the SACs on the next working day following the first acute consultation. See Table 1 for eligibility criteria.

**Table 1:** Eligibility criteria

Inclusion	Exclusion
Female 16 years or older Subjected to rape or attempted rape At least one SAC consultation within 3 days post rape or attempted rape Able to participate in the intervention within 14 days post rape Written consent	Men and trans persons Under 16 years of age Time since rape more than 3 days Not able to participate in the intervention within 14 days post rape Having cognitive disabilities Having other severe illnesses (acute psychosis, acute risk of suicide, serious drug and alcohol problems, other) Currently in PTSD treatment Ongoing violence or threats Total amnesia for the rape Do not speak Norwegian

Women who were eligible for inclusion and consented to further psychosocial support by SAC staff were recruited by a research assistant (RA) and given oral and written information about the study. The consenting women received a study ID and were directed to an electronic questionnaire for registering baseline data (background and other mental and physical health outcome data; see study protocol [25]. Thereafter, a structured PTSD interview (PSS-I-5) was scheduled by a trained clinical psychology student and was conducted by phone or video conference. A participant was deemed randomizable if critical baseline data had been collected, i.e., of written informed consent, completion of the questionnaire, and the clinical interview. The consenting participants also provided cortisol samples from hair and saliva as well as actigraphy recordings for a minimum of 3 days before being randomized by the RA.

### Intervention

#### Modified prolonged exposure therapy

In the intervention group, patients were assigned to a maximum of 5 weekly sessions of 60–90 min of mPE in addition to TAU, starting the mPE within 14 days post rape. The main procedures in mPE are psychoeducation about normal reactions to trauma, information on which factors can predispose to PTSD and maintain PTSD symptoms, and imaginal and in vivo exposure [28]. The exposure focuses on the specific trauma memories and associated trauma reminders. During imaginal exposure, traumatic memories are processed thoroughly to enhance adaptive responses and thoughts about the world and the self after the rape. The procedures are delivered systematically to enhance emotional engagement with the trauma memory, and to reduce avoidant behavior. The mPE sessions were audiotaped on a recorder device integrated into the PE Coach smartphone application [29]. Participants were encouraged to listen to the recordings between sessions for repeated exposures, see Table 2.

**Table 2:** Components of the mPE intervention

**Session 1**	Psychoeducation and rationale: common reactions to trauma, overall rationale of mPE and imaginal exposure Targeting index trauma Repeated imaginal exposure and processing Assigning homework: listen to recording
**Session 2**	Rationale of in vivo exposure Making an in vivo exposure hierarchy Repeated imaginal exposure and processing Assigning homework: In vivo exposure and listen to recording
**Session 3**	Repeated imaginal exposure and processing Assigning homework: In vivo exposure and listen to recording
**Session 4**	Repeated imaginal exposure and processing Assigning homework: In vivo exposure and listen to recording
**Session 5 (or last session)**	Repeated imaginal exposure and processing Discussing changes and improvements

### Treatment as usual

The TAU group received the standard care that is routinely provided at the SACs. This included psychosocial counseling around topics relevant to the individual subject, psychoeducation, practical advice about daily functioning, feedback regarding test results for sexually transmittable diseases, advice on whether to file a police report, etc. TAU is delivered in an unsystematic manner and varies across centers, and there is no standard for the length and number of sessions. TAU is provided either physically on site, or by telephone.

### Sample size

As we are reporting an internal pilot study, a formal sample size calculation was not undertaken. We already had power-estimated the required sample size for the main trial, indicating a need for ~ 185 patients [25]. Instead, we examined the recruitment pace during the 6-month pilot study period to indicate the possibility of reaching the targeted sample size for a full-scale RCT within the planned timeframe. We aimed to recruit and randomize at least 10% of all the patients who visited the SACs in the internal pilot period, which agrees with the findings of Cocks et al. [17], who recommend a sample size of at least 9% of the main trial sample size.

### Randomization

Randomization was performed in a 1:1 ratio by the RA using a computer-generated allocation sequence. The randomization was stratified with centers as the stratifying variable and variable block sizes were used to ensure equal group sizes and help conceal group allocations.

### Blinding

Due to the nature of the interventions, blinding of patients and therapists was not possible, but the outcome assessors were kept blind to the allocation. Efficacy data will be kept blinded for the trial project management and statistician until the collection of primary outcomes (3 months post-trauma) in the main RCT is completed.

### Setting

Recruitment took place between June 13^th^, 2022, and December 13^th^, 2022. Trondheim started recruiting in June, Oslo in October, and Vestfold in November. The training of PE therapists took place between January and September 2022. The training of research assistants, site coordinators, and psychology students was also conducted throughout 2022.

### Outcomes

#### Primary outcome (feasibility)

Feasibility was determined by (i) the number of eligible participants recruited to the trial, (ii) the retention rate, (iii) the training of therapists in mPE to deliver the intervention, (iv) successfully detecting harms, and (v) the application cortisol measures and actigraphy.

This study would be deemed feasible if ≥ 10% of eligible participants were successfully recruited to the trial, ≥ 60% of the recruited participants completed the final assessments (3 months post-trauma), the therapists delivered the intervention with “good” or “excellent” adherence, the harm detecting system detected and reported adverse and serious adverse events (AEs and SAEs), and if ≥ 50% of participants were willing to collect biological measures and wear actigraphy.

#### Secondary outcome (clinical outcome measures)

Outcome measures in this pilot study were examined at baseline, post-treatment, and 3 months post trauma. The full details of assessment measures in the main trial have been published elsewhere [25].

##### PTSD symptom scale interview (PSS-I-5)

The PSS-I-5 is a 24-item semi-structured interview for assessing PTSD symptoms in the past month and makes a diagnostic determination based upon DSM-5 criteria for PTSD. Questions assess for frequency and intensity of 20 DSM-5 PTSD symptoms, and symptom items are rated on a 5-point Likert scale of frequency and severity ranging from 0 (“not at all”) to 4 (“six or more times a week/ severe”). The sum of the 20 items yields a total PTSD symptom severity score, ranging from 0 to 80, indicating a probable PTSD diagnosis. The PSS-I-5 is a valid and reliable instrument for assessing PTSD diagnosis and severity [30]. We used a translation and modified version that has not been validated in a Norwegian population.

##### PTSD symptom checklist (PCL-5)

The PCL-5 is a 20-item self-report instrument that assesses symptoms of PTSD according to DSM-5. The items are rated on a 5-point Likert scale (0 = “not at all” to 4 = “extremely”), resulting in a total score between 0 and 80. The PCL-5 has a variety of purposes, including monitoring symptom changes during and after treatment, screening individuals for PTSD, and making a provisional PTSD diagnosis, and has demonstrated excellent reliability and validity in different trauma populations [31]. We used a translation that has not been validated in a Norwegian population.

##### Patient Health Questionnaire (PHQ-9)

The PHQ-9 is based on the DSM-4 diagnostic criteria for major depressive disorder and has remained unchanged in the DSM-5 update. It uses nine items to assess and monitor the severity of depression symptoms during the last 2 weeks. Participants self-reported on a 4-point Likert scale (0 = “not at all” to 3 = “nearly every day”), yielding a total score range of 0–27, with cut-off scores between 8 and 11 to detect clinical depression [32, 33]. The PHQ-9 has been widely validated [34].

##### General Anxiety Disorder (GAD-7)

The GAD-7 is a seven-item self-report instrument used to assess the DSM-IV diagnostic criteria for generalized anxiety disorder (GAD). Participants are asked how often during the last 2 weeks they have encountered anxiety symptoms on a scale from 0 to 3 on a 4-point Likert scale (0 = “not at all” to 3 = “nearly every day”), resulting in a total score between 0 and 21, with cut-off scores between 7 and10 for identifying GAD [35]. The GAD-7 is valid for screening for generalized anxiety disorder and assessing its severity in clinical practice [36].

### Biological assessments

We collected samples of the steroid cortisol from both saliva and hair. A small hair sample (strands of 3 mm in diameter) was collected from the participant`s scalp, packed into an aluminum foil for storage at the Biobank1®, Trondheim, and sent for analyses at the laboratory of Prof. Dr. Kirschbaum, Technische Universität Dresden, Germany. Hair steroid analysis was performed using the liquid chromatography-tandem mass spectrometry (LCMS/MS). Liquid chromatography separates different components in a mixture, and mass spectrometry identifies each separated component.

Participants received test tubes and instructions on how to collect saliva and store samples at home. Three samples were collected 3 days in a row: at their morning wakeup, after 30 min, and before going to bed at night, altogether nine samples. The samples were analyzed at the Dept. of Medical Biochemistry, St. Olav’s Hospital, Trondheim, with liquid chromatography-tandem mass spectrometry (LC–MS/MS) after liquid–liquid extraction. The limit of quantitation was 0.50 nmol/L.

### Physical activity and sleep

We measured physical activity and sleep for seven consecutive days using two AX3 (Axivity, Ltd., UK) accelerometers attached to the skin on the right thigh and lower back. The sensor streams were analyzed using a machine learning model [37, 38], which achieved an overall accuracy of 95% in detecting the time spent sitting, standing, walking, running, cycling, lying down, and sleeping.

### Data collection and storage

Patient self-report questionnaire data were collected using an electronic survey system, administered by the Clinical Research Unit at St. Olavs University Hospital and NTNU. Participants received an email and SMS with links to the questionnaire after providing written consent. To access the questionnaire, respondents had to use a secure login via BankID (a Norwegian Bank identifier code). The research coordinators at each study site collected medical record data regarding the assault and relevant clinical information. The RA collected practical monitoring data. The data were plotted into a web-based data collection system (web-CRF3) developed and administered by the Clinical Research Unit, NTNU/St. Olavs Hospital. The information was deidentified and encrypted. Similarly, all steps regarding mPE and TAU were collected by the therapists performing the treatment and entered into the same web-CRF3 system.

All participants received a unique participant’s ID number, which was stored with the highest possible level of security in a separate research file area provided by the services of sensitive data (TSD) at the University of Oslo. The data were then exported from encrypted files and stored in separate secure areas provided by the Data Protection Official at the St. Olavs Hospital. Trial data are stored until the completion of analysis, (data collection estimated to be finished by Dec 31, 2025), and then deleted after at least 5 years.

### Data analyses

The analysis of the data was conducted from March to June 2023, using SPSS version 28. Descriptive statistics (means, standard deviations, and proportions) were used to describe the demographic and clinical characteristics of the participants and feasibility outcomes. Statistical analyses to determine significant differences were not conducted due to the small sample size and the pilot nature of the project.

## Results

The results are reported using the CONSORT extended guidelines for pilot and feasibility trials [26]. In line with these recommendations, we did not evaluate the preliminary treatment efficacy. The main aim of the internal pilot study was to make an informed assessment of the feasibility of the planned full-scale randomized trial. Care should be taken when interpreting the results of this trial due to the small number of participants.

### Baseline characteristics

All participants were females aged between16 and 52 years, with a mean age of 21.7 years. Regarding occupational status, 16 (72.7%) participants were students. All except one participant reported being forcibly raped, including penetration, and two participants reported incapacitated rape. As many as 12 women (54.5%) reported that the assailant was someone they had recently met (within the last 24 h), and six (27.3%) reported being raped by a partner or friend. As many as 20 participants (90.9%) reported prior traumatization, whereas nine (40.9%) of these participants had experienced prior sexual assault (see Table 3).

**Table 3:** Baseline demographic and clinical characteristics

Baseline characteristics (*N* = 22 women)	*N*	%
**Age** (16–52 years, mean = 21.7 (SD 7.4), median = 20 (IQR 18–22)		
** Occupational status**		
Employed	**5**	**22.7**
Student	**16**	**72.7**
Other	**1**	**4.5**
** Assault location**		
Private	**15**	**68.2**
Public	**7**	**31.8**
** Relationship with assailant**		
Partner (current or ex) or friend	**6**	**27.3**
Acquaintance (recently met < 24 h)	**12**	**54.5**
Stranger/unknown	**4**	**18.2**
** Type of assault**		
Penetration (oral, vaginal, and anal)	**21**	**95.5**
Other (stranglehold, hitting, kicking, lifted, thrown)	**1**	**4.5**
** Bodily injury**		
No injury	**14**	**63.6**
Mild injury	**8**	**36.4**
Moderate	**1**	**4.5**
** Alcohol influence**		
No	**3**	**13.6**
Yes	**16**	**72.7**
Not reported	**3**	**13.6**
Suspicion of being drugged	**2**	**9.1**
** Prior traumatization (*n* = 21)**		
Yes	**20**	**95.2**
No	**1**	**4.8**
Number of traumas: range = 1–8, mean = 3.1		
Prior sexual assault (in childhood or as an adult)	**9**	**42.9**
Sexual assault in childhood	**5**	**23.8**

### Feasibility outcome

Progression criteria regarding the feasibility of the EIR-study protocol were defined before conducting this pilot study, see Table 4.

**Table 4:** Progression criteria

Feasibility outcome	Criteria	Criteria met?	Adjustments
Recruitment	- Minimum 10% eligible and consenting patients to proceed in the study within 6 months	Almost, 9.4%	- Widening inclusion criteria from 3 to 7 days - Training more therapists (*n* = 4) to increase capacity to include - Efforts to recruit more SACs
Retention	- Minimum 60% retention - Completion of main outcome questionnaires = 80%	Yes Yes	- Research assistants/coordinators contacts participants × 3 to improve retention
Intervention implementation	- Training personnel through workshops and supervision (*n* = 12) - Therapist’s adherence to protocol is scored “good” or” excellent”	Yes Yes	
Harm report system	- Detecting adverse and serious adverse events during the trial, managing reports of harm effectively - Establishing DMC	Yes Yes	
Applying biological measures and actigraphy	- More than 50% of the participants: - Consent to collect hair and saliva samples - Comply to sampling instructions - Consent to use actigraphy for 7 days - Logistics and analysis are viable	Yes No Yes Yes	- Instructions are made more accurate to ensure correct sampling

### The recruitment process

**Fig. 1:**
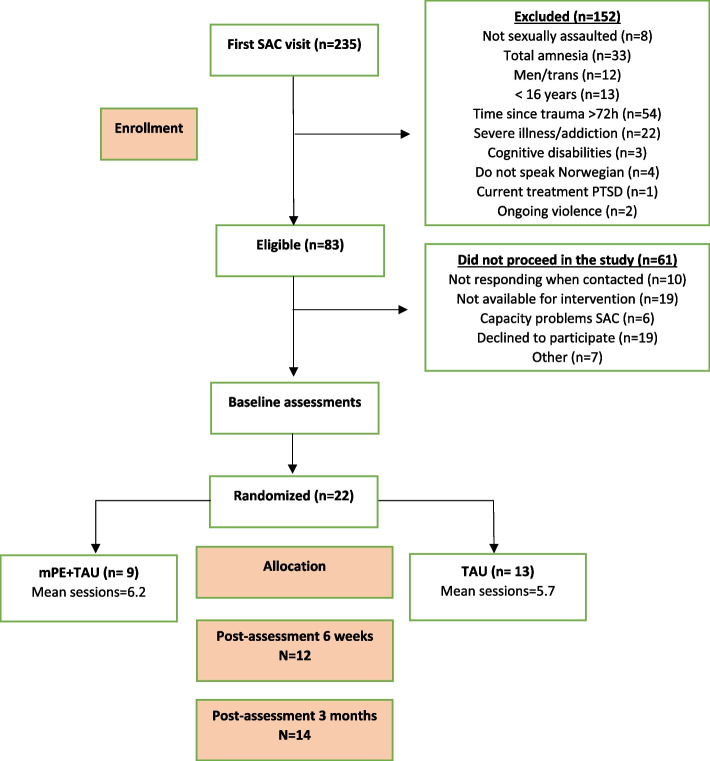
CONSORT flow-chart

Following eligibility screening of the 235 patients, 152 (64.7%) did not meet the inclusion criteria. Of the 83 (35%) eligible patients, 61 (26%) declined or did not participate for other reasons, and 22 (9.4%) were included in the study. As shown in Fig. 1, nine participants were allocated to the intervention group (mPE + TAU), and 13 women to the control group (TAU). See Fig. 1.

Recruitment was initially slow due to start-up delays at two of the SACs. The recruitment rate for the total period was 9.4%, which is slightly less than our pre-defined goal of 10%. In October, Oslo began to recruit, and as the largest SAC, the recruitment pace increased significantly.

There were also challenges with recruitment during holidays, as the participants were sometimes unable to attend mPE sessions within 14 days; therefore, they were not included in the study. Periodically, the recruitment pace suffered some lags as therapists reached their maximum case load.

**Fig. 2:**
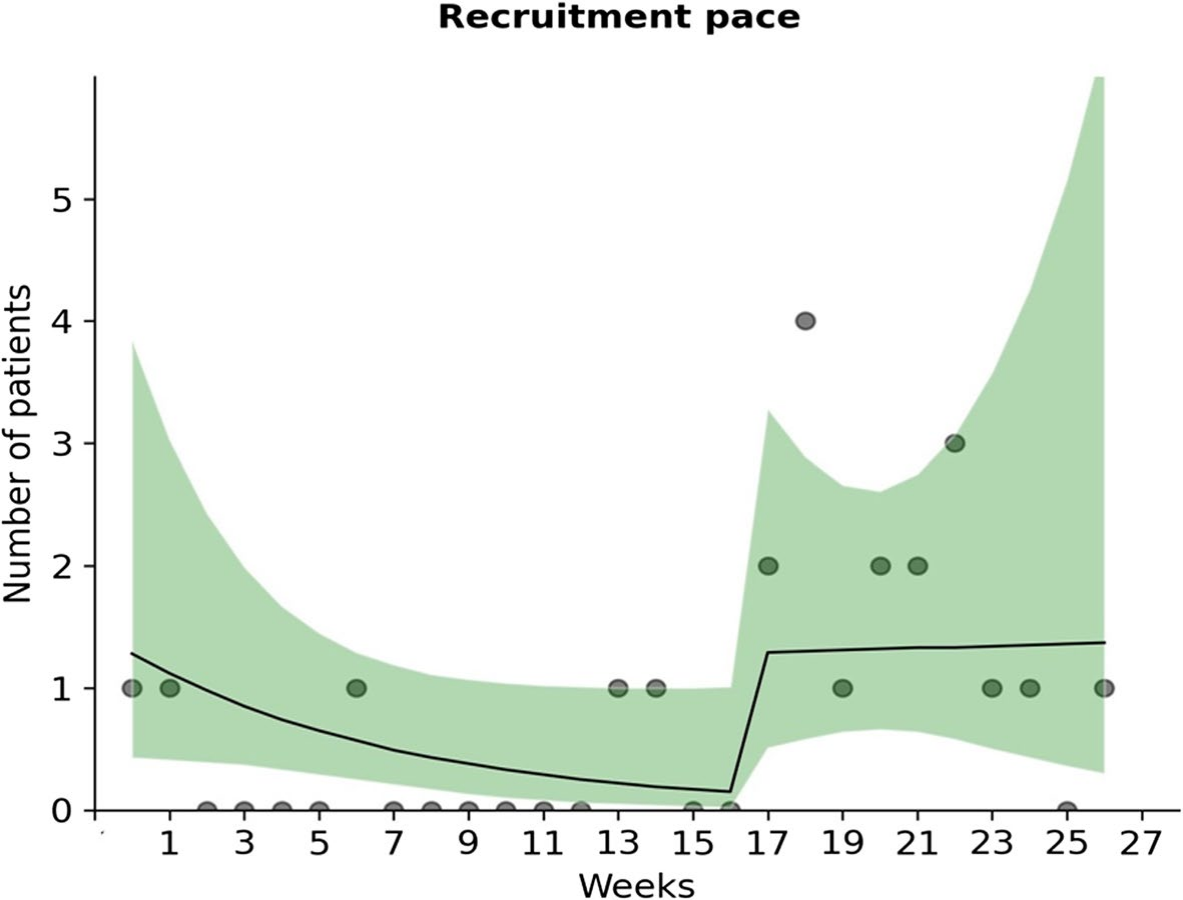
A segmented line portrays the recruitment pace most realistically as it correctly captures the recovery of the recruitment speed during the second half of the pilot-study period (the shaded area is the 95% confidence interval, and the dots are the observed counts)

We fitted a regression count model with a negative binomial distribution (to accommodate for overdispersion) for predicting the recruitment pace based on the observed time trend. We fitted a linear (SSE = 23.3), nonlinear (SSE = 23.4), and a segmented line (SSE = 18.9), of which the segmented line deviated the least from the observed counts when comparing the SSE (sum of squared errors). Moreover, the segmented line did not portray an unrealistic growth at the end of the recruitment period (see Fig. 2), as did the other lines. This line indicated a relatively stable pace of ~ 1.3 patients per week during the second half of the pilot-study period, which indicates that an additional ~ 125 weeks (or ~ 2.4 years) are needed to reach a target of 185 patients, see Fig. 2.

Several adjustments were made to optimize recruitment. Four extra therapists (three mPE therapists and one TAU therapist) were recruited and trained to increase capacity. The inclusion criteria were widened from 3 to 7 days after rape but still provided that the intervention had to start within 14 days after the rape incident. The case report forms were simplified and shortened to reduce the workload on the TAU therapist’s behalf, and coordinators’ data collection from the medical records was reduced, as we found that some items were covered by self-report questionnaires. There is also an ongoing process of recruiting one or two more SACs.

### Retention

All 22 participants (100%) completed assessments at baseline, and 14 (63.6%) completed the final assessment at 3 months. Six participants (27.3%) did not complete final assessments.

The intervention group received between three and 11 consultations (mean = 6.2) of mPE + TAU, compared to one to nine consultations (mean = 5.7) in the TAU group. Completion in the intervention group was defined as at least three sessions of mPE.

The six participants who did not complete the final assessments were not considered to have dropped out of treatment, as they received between three and seven (mean = 5.3) consultations of either mPE + TAU or TAU.

Participants dropped out of assessments and/or treatment for various reasons: two participants reported that the follow-up treatment did not meet their expectations or was too time-consuming, and one had a significant commute to the SAC. Three participants did not show up for treatment and did not answer the phone when contacted. Since all of them had an average of 5.3 consultations, it could also be considered that they had completed or partly completed treatment. There was no definition of completion in the TAU group.

Regarding the completion of questionnaires and missing data, all 22 participants completed all items on the PCL-5 and GAD-7, and 21 completed the PHQ-9 at baseline. All 22 participants completed the PSS-I-5 interview. At 3 months, 14 participants completed both the PCL-5, GAD-7, and PHQ-9 and the PSS-I-5 interview.

To enhance the retention and completion of questionnaires, we gave participants gift cards if they completed the assessments at baseline, 6 weeks, and 3 months, and travel expenses were refunded.

### Intervention implementation

Therapists (*n* = 12) who received training in the mPE procedure were experienced SAC employees with various educational backgrounds (psychiatric nurses, social workers with therapy training). All completed training with a mandatory 4-day workshop consisting of education about the theoretical underpinnings of PE, as well as hands-on training in the use of PE and the mPE protocol through lectures, videos, roleplay, and self-studies. The training was led by the first author, which is a certified PE supervisor and trainer. Supervision was provided regularly and upon request from the therapists. To avoid contamination, the therapist delivering TAU was not trained in mPE. Treatment adherence forms used in previous clinical trials with PE were adapted to fit the mPE intervention procedure to report which treatment components they included in the sessions, and to what extent they included components not part of the intervention (Foa E, Kushner E, Capaldi S, Yadin E: Manual for adherence ratings for prolonged exposure therapy, unpublished). In addition, all mPE sessions were audio-recorded for assessing adherence. mPE therapists completed adherence checklists after each session, and approximately 30% (*n* = 7) of the audio-recordings were selected in a random fashion and assessed by TH to monitor protocol adherence. Adherence was assessed on a scale from “poor,” “barely adequate,” “good,” and “excellent,” according to how many required elements were included in each session. Out of the seven audiotapes assessed, all were scored as “excellent”, meaning that the therapists had completed all or almost all (minus one) session elements. How participants and therapists experienced mPE will be explored and elaborated in a separate study using qualitative interviews.

### Harm report system

The SAC staff were instructed to monitor adverse events (AEs) and serious adverse events (SAEs) that participants experienced during the trial and to fill out web-CRF3 if present. Members of the research group were alerted automatically by e-mail within a few hours when an AE or SAE was registered. AEs were defined as any undesirable experience experienced by participants during the trial. SAEs were defined as death, suicide, attempted suicide, serious self-harm, acute psychosis or mania, severe intoxication, or other psychological or somatic illnesses or conditions that demanded acute treatment (epilepsy, heart attack, stroke). After clinical considerations, AEs and SAEs were categorized according to whether they were perceived as related to the intervention or not. A data monitoring committee (DMC) consisting of a clinical psychologist, a statistician, and a senior project advisor was established prior to recruitment. Their mandate was to review the safety aspects of the trial and the validity and integrity of the collected data. In the case of SAEs, DMC was to be granted access to information regarding treatment conditions to determine whether the adverse event was likely related to the treatment and whether the trial should be stopped.

Two participants reported on suicidal ideation, eliciting reports of SAEs. One patient reported intoxication with an uncertain amount of medication after session four, allegedly as a response to long-lasting relationship problems within the family. The research group investigated both cases immediately after reports of SAE and found that both participants were properly cared for before the SAEs were reported (both were assessed for suicide risk and the need for further psychiatric care by a medical doctor at the Emergency Department). The research team also consulted with health professionals who had worked with the two participants and collected medical records for additional information. Finally, the research team discussed whether the SAEs were related to the intervention. Both participants had a history of suicidal ideation, and one had also previously been admitted for suicidal behavior. Both had experienced prior traumatization and sexual assault. None of the participants stated that treatment at the SAC elicited the suicidal ideation/behavior, nor did the staff who met the participants. In both cases, the research team concluded that the suicidal ideation and suicidal behavior were most likely a result of previous and recent trauma and cumulative stress, young age, and a history of suicidal ideation and suicidal behavior before the recent trauma. Both young age and recent rape are known risk factors for suicidal ideation and suicidal behavior [39, 40]. Neither of the two participants were excluded from the study, but both declined further psychosocial treatment due to practical reasons (distance, school).

In addition, 13 adverse events were reported, including minor adverse effects (itching and rash from the use of the accelerometer tape) and deviations from the actigraphy and cortisol sampling instructions (the accelerometers fell off, an incomplete number of saliva samples, samples not refrigerated, etc.), as shown in Table 5. We concluded that the feasibility of the harm reporting system was good.

**Table 5:** Adverse events and serious adverse events

	**Adverse events**	**Serious adverse events**
Rash from accelerometer tape	3	
Actigraphy not used according to instructions	3	
Saliva samples not taken according to instructions	7	
Suicidal rumination		2
Attempted suicide		1

### Applying cortisol measuring methods and actigraphy

All 22 participants consented to sample hair cortisol at baseline, 13 (59.1%) post-treatment, and 12 (54.5%) at 3 months. Data from the hair samples are not yet analyzed but will be presented when results from the main trial are disseminated.

All 22 participants consented to saliva cortisol collection at baseline, 13 (59.1%) post-treatment, and 12 (54.5%) at 3 months. The collected morning samples showed the expected diurnal variation and cortisol awakening response (CAR). The analytical coefficient of variation was 5.0%.

Four participants had night samples showing an increased level of saliva cortisol. Of these, only one participant had increased saliva cortisol levels in all three-night samples, and she was informed by the RA about the increased levels, for further check-up with her general practitioner.

Only four participants collected all nine samples correctly. Collecting saliva samples at specific timepoints was challenging for some of the participants, and some were reluctant to refrigerate the samples because they had not disclosed the recent trauma to their cohabitants. Although the level of missing data was high, repeated measurements compensated to some extent for the missing values. However, we decided to increase the participants´ sampling compliance by revising the instruction brochure for the participants.

According to Biobank1®, the logistical process of sampling and shipping of saliva tests was good. The samples were submitted according to plan, and only two samples were not processable, due to insufficient amounts of saliva.

All but one participant (95.5%) agreed to wear actigraphy at baseline, 12 (57.1%) agreed post-treatment, and 10 (47.6%) agreed at 3 months. Data from the accelerometers are not yet analyzed but will be presented when results from the main trial are disseminated.

We tried to schedule the biological measurements at the same time as they had treatment consultations at the SAC to reduce the number of visits. Although participants seemed to tolerate the biological measurements, and we were able to collect meaningful data from them, the research team has decided to continuously evaluate the cost–benefit ratio of including biological measures in the main trial.

### Clinical outcome

At baseline, the mean score of self-reported post-traumatic stress symptoms (PCL-5) for all participants was 47. For the clinician-administered PTSD interview (PSS-I-5), the mean score was 36.4. For depression (PHQ-9), participants scored a mean of 16.8, and for anxiety (GAD-7), participants scored a mean of 12.9.

All but two participants scored above the cut-off for PTSD on the PCL-5, and all but four scored above the cut-off after being assessed by a clinician with the PSS-I-5. All but three participants scored above the cut-off for moderate to severe depression and 15 participants scored above the cut-off for moderate anxiety. This indicates an overall high symptom load within the first 2 weeks after the rape incident, see Table 6.

**Table 6:** Baseline assessments

	***N***	**Mean**	**SD**	**95% CI**	**Cut-off**
Total score PCL-5	22	47	12.8	41.5–52.4	31–33
Total score PSS-I-5	22	36.4	14.1	30.7–42.2	23
Total score PHQ-9	21	16.8	4.8	14.7–18.8	8–11
Total score GAD-7	22	12.9	4.8	10.8–14.9	7–10

## Discussion

### Feasibility

The use of an internal pilot was an efficient way to determine feasibility and optimize trial processes. The findings demonstrate that the pre-defined progression criteria were mostly met or could be met with certain refinements and support the feasibility of a full-scale RCT. We made several adjustments to the procedures, but not to the extent that the original design was compromised.

### Strengths and limitations

This study aimed to mimic the real-life clinical conditions. We trained SAC nurses, social workers, and other health professionals at the centers as therapists responsible for delivering the intervention. If an evidence-based intervention such as mPE can be implemented in Norwegian SACs and delivered by nurses and social workers, this could improve the generalizability of the findings considerably.

Measures were taken to decrease contamination between the two conditions, but it was challenging to map all the components of the TAU. The Norwegian guidelines for psychological support at the SACs are vague [23], and consequently, all three SACs had different TAU routines. Therefore, TAU is not operationalized in this study. The therapists who received training in mPE were accustomed to performing TAU before participating in the study, and due to capacity problems, they occasionally had to take care of participants allocated to TAU. This could compromise the internal validity of the study. Additionally, other types of support from outside the SACs were not recorded or controlled for, and therefore, may have influenced the outcomes in various ways.

Approximately 10% of rape survivors seek help [10]. A study by Vik et al. [41] revealed that 59% of Norwegian SAC patients have at least one vulnerability factor, and 29% have more than one factor. Vulnerability was defined as having an intellectual or physical disability, a history of mental health problems, alcohol or substance abuse, or a prior sexual assault. The demographic characteristics of our sample correspond with Vik’s findings in several aspects: 95.2% of our sample reported prior traumatization, with 42.9% experiencing previous sexual trauma. Additionally, 9.4% of the patients were excluded due to severe mental illness and/or addiction. Only 1.3% of potential participants were excluded due to cognitive disabilities. Moreover, 1.7% were excluded for not speaking Norwegian, which could lead to minority groups, which are typically considered vulnerable, being underrepresented in the study. Although these exclusion criteria might result in a somewhat selected group, Vik's study indicates that our sample is representative for Norwegian SAC patients.

Young age and past victimization increase women’s risk of being raped [42]. In this study, we found that the mean age was 21.7 years. Altogether, 81.8% of the participants met or knew about the perpetrator before the assault. This corresponds with a recent Norwegian population study [10] and research on female victims of sexual assault in Denmark [43].

Research shows that seeking help shortly after rape is associated with better health and fewer symptoms [44, 45]. Receiving emphatic, trauma-informed care from more formal sources, including sexual assault nurse examiners, and personnel at SACs, can reduce post-traumatic stress symptoms and facilitate recovery [46, 47]. It is therefore important that efficient health care services are available for people subjected to rape, and that these services are known to the public. Unfortunately, many victims of sexual assault never seek medical attention, and sexual violence is underreported to both health care providers and police.

A common reaction to trauma, and one of the core symptoms of PTSD is avoidance (e.g., avoiding thinking or talking about the trauma, avoiding trauma reminders). It is possible that the most avoidant women hesitate to visit SACs. The active confrontation with trauma memory and reminders might also explain why only 9.4% of the proportion of eligible patients consented to participate in the study.

Two participants reported on suicidal ideation, and one reported on suicidal behavior, which was reported efficiently through the harm reporting system. Both were very young and had a history of sexual assault and suicidal ideation. The research team discussed with DMC whether we should interpret the two SAEs as related to study participation or not. The DMC concluded, after going through medical records and relevant documentation, that there was a very low probability that the incidents were related to the study or intervention, that the study was ethically sound, and that there was no indication for terminating the trial. The DMC recommended to include monitoring of suicidal ideation/behavior through the treatment, but not to change inclusion criteria (e.g., systematically excluding more patients due to suicidal ideation). Given the physical environment at each SAC (open 24 h, open door policy, medical staffing, etc.), and the fact that all participants were de facto help-seeking individuals who consented to participate, we concluded that having suicidal ideation or prior suicidal behavior as exclusion criteria would restrict valuable research on this population. Although there is no systematic suicide risk assessment in this study protocol, this is a systematic and integrated procedure at hospitals and emergency departments in Norway.

A limitation of this study is the exclusion of substance abusers, minority women not speaking Norwegian, men, and transgender persons. These might be subgroups in which even more than the general population should be targeted for interventions.

The assessments at five different time points could seem too much of a workload for the participants, thereby hindering recruitment and leading to premature drop-out from the study. We have added to the protocol that only certain questionnaires and interview data will be mandatory for participation in the study, and that the cortisol sampling and actigraphy measurements will be optional.

### Conclusion and further research

Our conclusion is that it is feasible to proceed to a full-scale RCT of early intervention after rape that compares mPE + TAU to TAU alone. Minor design refinements were made to enhance the main study outcome. Careful consideration should be taken regarding effective recruitment strategies, participation workload, and monitoring of adverse events.

## Data Availability

Anonymized and analyzed data and statistical codes will be available from the corresponding author on reasonable request, as is the full protocol.

## Abbreviations

EIR: Early Intervention after Rape
PTSD: Posttraumatic stress disorder
PE: Prolonged exposure therapy
mPE: Modified prolonged exposure
SAC: Sexual assault center
TAU: Treatment as usual
RCT: Randomized controlled trial
CONSORT: Consolidated Standards of Reporting Trials
RA: Research assistant
PSS-I-5: PTSD Symptom Scale Interview for DSM-5
DSM: Diagnostic and Statistical Manual of Mental Disorders
PCL-5: PTSD Symptom Checklist for DSM-5
PHQ-9: Patient Health Questionnaire-9
GAD-7: General Anxiety Disorder-7
LCMS/MS: Liquid chromatography–mass spectrometry
CAR: Cortisol awakening response
AX3: 3-Axis accelerometer
Bank-ID: Bank identifier
CRF3: Case report form
TSD: Tjenester for Sensitive Data (Services for Sensitive Data)
SPSS: Statistical Package for the Social Sciences
DMC: Data Monitoring Committee
AE: Adverse event
SAE: Serious adverse event
